# Supplementary material for: Polyketide synthase gene clusters in the ericoid mycorrhizal fungus Oidiodendron maius: genome-wide prediction, expression profiling, and functional characterization of knockout mutants
Source: Front Plant Sci. 2026 Sep 16;17:1924430. doi: 10.3389/fpls.2026.1924430 (PMC13623903; doi:10.3389/fpls.2026.1924430)
Supplement: Supplementary file 1 [file SupplementaryFile1.zip › Supplementary Materials/Supplementary Folder 1.zip!/Oidma1_AssemblyScaffolds_Repeatmasked/knownclusterblast/cluster2/jgi.p_Oidma1_71694_mibig_hits.html]

| MIBiG Protein | Description | MIBiG Cluster | MiBiG Product | % ID | % Coverage | BLAST Score | E-value |
| --- | --- | --- | --- | --- | --- | --- | --- |
| ABM91454.1 | lysergyl\_peptide\_synthetase\_LpsB | BGC0000348 | Ergovaline\_biosynthetic\_gene\_cluster | 36 | 79.4186046512 | 396 | 7.9e-110 |
| ctg1\_orf003 |  | BGC0000334 | Cyclosporin\_biosynthetic\_gene\_cluster | 34 | 80.0 | 392 | 1.1e-108 |
| EAL89049.1 | nonribosomal\_peptide\_synthase,\_putative | BGC0000355 | Fumiquinazolines\_biosynthetic\_gene\_cluster | 33 | 80.3488372093 | 377 | 4.9e-104 |
| ACZ66258.1 | APS1 | BGC0000304 | Apicidin\_biosynthetic\_gene\_cluster | 29 | 106.744186047 | 372 | 1.2e-102 |
| BAO73252.1 | non-ribosomal\_peptide\_synthetase | BGC0000456 | Verlamelin\_biosynthetic\_gene\_cluster | 31 | 87.0930232558 | 372 | 1.2e-102 |
| AAA33023.2 | HC-toxin\_synthetase | BGC0000370 | HC-toxin\_biosynthetic\_gene\_cluster | 30 | 106.511627907 | 364 | 4.3e-100 |
| ACR78148.1 | BSLS | BGC0000312 | Bassianolide\_biosynthetic\_gene\_cluster | 32 | 78.2558139535 | 363 | 5.7e-100 |
| AGC83576.1 | NRPS | BGC0000818 | Notoamide\_biosynthetic\_gene\_cluster | 33 | 79.7674418605 | 358 | 3.1e-98 |
| ctg1\_orf00001 |  | BGC0000901 | Ferrichrome\_biosynthetic\_gene\_cluster | 34 | 79.7674418605 | 357 | 4.1e-98 |
| ctg1\_orf000000 |  | BGC0000901 | Ferrichrome\_biosynthetic\_gene\_cluster | 33 | 74.5348837209 | 312 | 2e-84 |
| CAP93139.1 |  | BGC0000357 | Fungisporin\_biosynthetic\_gene\_cluster | 32 | 84.5348837209 | 354 | 3.4e-97 |
| OAQ83772.1 | nonribosomal\_peptide\_synthase | BGC0001358 | Leucinostatins\_biosynthetic\_gene\_cluster | 32 | 83.8372093023 | 353 | 5.8e-97 |
| AGQ43600.1 | HC-toxin\_synthetase | BGC0001166 | HC-toxin\_biosynthetic\_gene\_cluster | 32 | 81.976744186 | 349 | 8.4e-96 |
| AGA37267.1 | NRPS | BGC0000816 | Malbrancheamide\_biosynthetic\_gene\_cluster | 34 | 78.2558139535 | 348 | 1.9e-95 |
| CAA79245.2 | enniatin\_synthetase | BGC0000342 | Enniatin\_biosynthetic\_gene\_cluster | 32 | 78.6046511628 | 348 | 1.9e-95 |
| EFY94500.1 | non-ribosomal\_peptide\_synthetase | BGC0000337 | Destruxins\_biosynthetic\_gene\_cluster | 31 | 79.3023255814 | 348 | 2.5e-95 |
| AMM63162.1 | AniA | BGC0001371 | Echinocandin\_B\_biosynthetic\_gene\_cluster | 33 | 81.2790697674 | 343 | 7.9e-94 |
| ADY16697.1 | TqaA | BGC0001142 | Tryptoquialanine\_biosynthetic\_gene\_cluster | 31 | 81.6279069767 | 337 | 4.3e-92 |
| EAW16180.1 | nonribosomal\_peptide\_synthase,\_putative | BGC0000293 | Acetylaszonalenin\_biosynthetic\_gene\_cluster | 31 | 77.7906976744 | 337 | 4.3e-92 |
| BAH23995.1 | nonribosomal\_peptide\_synthetase | BGC0000356 | Fumitremorgin\_biosynthetic\_gene\_cluster | 31 | 80.1162790698 | 336 | 1.3e-91 |
| ADM34138.1 | non-ribosomal\_peptide\_synthetase | BGC0001084 | Notoamide\_/\_stephacidin\_biosynthetic\_gene\_cluster | 34 | 77.7906976744 | 333 | 8.2e-91 |
| CRG85572.1 | nonribosomal\_peptide\_synthase,\_putative | BGC0001402 | Cyclochlorotine\_biosynthetic\_gene\_cluster | 32 | 81.3953488372 | 331 | 4.1e-90 |
| EPE34341.1 | Acetyl-CoA\_synthetase-like\_protein | BGC0001035 | Pneumocandin\_biosynthetic\_gene\_cluster | 30 | 80.2325581395 | 328 | 2.6e-89 |
| BAN91483.1 | putative\_Aculeacin\_A\_nonribosomal\_peptide\_synthase | BGC0001220 | Aculeacin\_A\_biosynthetic\_gene\_cluster | 30 | 81.0465116279 | 323 | 8.5e-88 |
| CBF87069.1 | nonribosomal\_peptide\_synthase,\_putative\_(Eurofung) | BGC0001290 | Emericellamide\_A\_/\_emericellamide\_B\_biosynthetic\_gene\_cluster | 31 | 81.3953488372 | 321 | 3.2e-87 |
| XP\_001227984.1 | hypothetical\_protein | BGC0001240 | Serinocyclin\_A\_/\_serinocyclin\_B\_biosynthetic\_gene\_cluster | 32 | 77.7906976744 | 321 | 4.2e-87 |
| ACI30655.1 | BEAS\_beauvericin\_nonribosomal\_cyclodepsipeptide\_synthetase | BGC0000313 | Beauvericin\_biosynthetic\_gene\_cluster | 31 | 77.7906976744 | 320 | 7.2e-87 |
| ABB90279.1 | non-ribosomal\_peptide\_synthetase | BGC0001057 | Zearalenone\_biosynthetic\_gene\_cluster | 31 | 83.023255814 | 319 | 1.2e-86 |
| AFN69082.1 | nonribosomal\_peptide\_synthetase\_6 | BGC0001249 | Dimethylcoprogen\_biosynthetic\_gene\_cluster | 31 | 78.3720930233 | 318 | 2.1e-86 |
| AFT91378.1 | EcdA | BGC0000338 | Echinocandin\_B\_biosynthetic\_gene\_cluster | 29 | 80.3488372093 | 312 | 2e-84 |
| CAP96445.1 |  | BGC0000420 | Roquefortine\_C\_/\_meleagrin\_biosynthetic\_gene\_cluster | 30 | 80.5813953488 | 305 | 1.8e-82 |
| ACJ04424.1 | aureobasidin\_A1\_biosynthesis\_complex | BGC0000307 | Aureobasidin\_A1\_biosynthetic\_gene\_cluster | 31 | 74.0697674419 | 302 | 2e-81 |
| AAF01762.1 | AM-toxin\_synthetase | BGC0001261 | AM-toxin\_biosynthetic\_gene\_cluster | 29 | 79.4186046512 | 295 | 1.4e-79 |
| CAI59267.1 | peptide\_synthetase\_3 | BGC0001263\_c2 | Ergometrine\_biosynthetic\_gene\_cluster | 30 | 80.5813953488 | 292 | 2.1e-78 |
| CBF76038.1 | nonribosomal\_peptide\_synthase,\_putative\_(Eurofung) | BGC0001399 | Fellutamide\_B\_biosynthetic\_gene\_cluster | 30 | 78.488372093 | 287 | 5.1e-77 |
| AHB82058.1 | non\_ribosomal\_peptide\_synthetase | BGC0001019 | Microsclerodermins\_biosynthetic\_gene\_cluster | 30 | 80.1162790698 | 269 | 1.5e-71 |
| CAD17792.1 | probable\_non\_ribosomal\_peptide\_synthetase\_protein | BGC0001363 | Ralsolamycin\_biosynthetic\_gene\_cluster | 29 | 78.6046511628 | 251 | 4.1e-66 |
| AHB82071.1 | non\_ribosomal\_peptide\_synthetase | BGC0001231 | Microsclerodermins\_biosynthetic\_gene\_cluster | 28 | 79.6511627907 | 250 | 9.1e-66 |
| AHJ31215.1 | Long-chain-fatty-acid--CoA\_ligase | BGC0000430 | Spumigin\_biosynthetic\_gene\_cluster | 26 | 79.5348837209 | 242 | 1.9e-63 |
| AGA37269.1 | NRPS | BGC0000819 | Paraherquamide\_biosynthetic\_gene\_cluster | 29 | 66.976744186 | 242 | 2.5e-63 |
| CDF96614.1 | Long-chain-fatty-acid--CoA\_ligase | BGC0001149 | Brabantamide\_biosynthetic\_gene\_cluster | 27 | 80.4651162791 | 241 | 3.2e-63 |
| AAZ03552.1 | McnC | BGC0000332 | Cyanopeptolin\_biosynthetic\_gene\_cluster | 29 | 81.511627907 | 240 | 9.4e-63 |
| AAY91420.2 | non-ribosomal\_peptide\_synthetase\_OfaB | BGC0000399 | Orfamide\_biosynthetic\_gene\_cluster | 28 | 77.0930232558 | 240 | 9.4e-63 |
| WP\_020635019.1 | hypothetical\_protein | BGC0001211 | Albachelin\_biosynthetic\_gene\_cluster | 30 | 76.0465116279 | 239 | 1.2e-62 |
| CAE15637.1 |  | BGC0001128 | Luminmide\_biosynthetic\_gene\_cluster | 29 | 76.7441860465 | 238 | 2.1e-62 |
| ACZ55942.1 | non-ribosomal\_peptide\_synthetase | BGC0000302 | Anabaenopeptin\_biosynthetic\_gene\_cluster | 29 | 75.6976744186 | 238 | 3.6e-62 |
| ABI26078.1 | OciB | BGC0000331 | Cyanopeptin\_biosynthetic\_gene\_cluster | 28 | 77.9069767442 | 235 | 2.3e-61 |
| ABI26077.1 | OciA | BGC0000331 | Cyanopeptin\_biosynthetic\_gene\_cluster | 27 | 79.0697674419 | 234 | 4e-61 |
| BAH22764.1 | nonribosomal\_peptide\_synthetase | BGC0001018 | Micropeptin\_biosynthetic\_gene\_cluster | 29 | 81.7441860465 | 234 | 5.2e-61 |
| CAJ34381.1 | NRPS\_protein | BGC0000445 | Thiocoraline\_biosynthetic\_gene\_cluster | 28 | 80.4651162791 | 233 | 8.8e-61 |
| CBZ42146.1 | putative\_non-ribosomal\_peptide\_synthetase | BGC0001117 | Himastatin\_biosynthetic\_gene\_cluster | 28 | 76.3953488372 | 233 | 1.2e-60 |
| AFP87549.1 | NrpS | BGC0001135 | Bicornutin\_biosynthetic\_gene\_cluster | 28 | 77.7906976744 | 232 | 1.5e-60 |
| AAY37655.1 | Amino\_acid\_adenylation | BGC0000437 | Syringomycin\_biosynthetic\_gene\_cluster | 28 | 79.4186046512 | 229 | 1.3e-59 |
| AJW76711.1 | DsaI | BGC0001196 | Desotamide\_biosynthetic\_gene\_cluster | 27 | 77.9069767442 | 229 | 1.7e-59 |
| BAH43871.1 | truncated\_linear\_pentadecapeptide\_gramicidin\_synthetase\_LgrC | BGC0000367 | Gramicidin\_biosynthetic\_gene\_cluster | 28 | 80.2325581395 | 229 | 1.7e-59 |
| AHD05614.1 | putative\_non-ribosomal\_peptide\_ligase/\_polyketide\_synthase\_hybrid | BGC0001033 | Paenilamicin\_biosynthetic\_gene\_cluster | 28 | 78.488372093 | 228 | 2.2e-59 |
| AEH59100.1 | amino\_acid\_adenylation\_domain-containing\_protein/NRPS | BGC0000385 | Lysobactin\_biosynthetic\_gene\_cluster | 27 | 80.8139534884 | 228 | 3.7e-59 |
| AAZ23078.1 | peptide\_synthetase | BGC0000291 | A54145\_biosynthetic\_gene\_cluster | 28 | 80.0 | 228 | 3.7e-59 |
| CAD17793.1 | probable\_non\_ribosomal\_peptide\_synthetase\_protein | BGC0001363 | Ralsolamycin\_biosynthetic\_gene\_cluster | 28 | 82.5581395349 | 227 | 4.8e-59 |
| AAO72425.1 | syringopeptin\_synthetase\_C | BGC0000438 | Syringopeptin\_biosynthetic\_gene\_cluster | 28 | 79.4186046512 | 227 | 6.3e-59 |
| AIW82285.1 | PuwH | BGC0001125 | Puwainaphycins\_biosynthetic\_gene\_cluster | 27 | 81.976744186 | 227 | 6.3e-59 |
| AEH41793.1 | HrmO | BGC0000374 | Hormaomycin\_biosynthetic\_gene\_cluster | 28 | 78.6046511628 | 226 | 1.1e-58 |
| ANI24100.1 | nonribosomal\_peptide\_synthetase | BGC0001235 | Antalid\_biosynthetic\_gene\_cluster | 27 | 80.6976744186 | 226 | 1.1e-58 |
| AAY37653.1 | Amino\_acid\_adenylation | BGC0000437 | Syringomycin\_biosynthetic\_gene\_cluster | 28 | 79.4186046512 | 225 | 2.4e-58 |
| CAB38518.1 | CDA\_peptide\_synthetase\_I | BGC0000315 | Calcium-dependent\_antibiotic\_biosynthetic\_gene\_cluster | 29 | 78.488372093 | 224 | 5.4e-58 |
| CBJ90082.1 | Non\_Ribosomal\_peptide\_synthetase\_(-succinylbenzoate--CoA\_ligase) | BGC0001132 | Xenotetrapeptide\_biosynthetic\_gene\_cluster | 27 | 82.3255813953 | 223 | 9.1e-58 |
| AEU11006.1 | NpnB | BGC0001029 | Nostophycin\_biosynthetic\_gene\_cluster | 27 | 80.5813953488 | 223 | 1.2e-57 |
| AFH75329.1 | nonribosomal\_peptide\_synthetase | BGC0000398 | Orfamide\_biosynthetic\_gene\_cluster | 27 | 74.7674418605 | 222 | 1.6e-57 |
| AED90002.1 | non-ribosomal\_peptide\_synthetase\_ThaA | BGC0000443 | Thanamycin\_biosynthetic\_gene\_cluster | 28 | 83.488372093 | 222 | 1.6e-57 |
| CAN89656.1 | putative\_non-ribosomal\_peptide\_synthetase | BGC0001070 | Kirromycin\_biosynthetic\_gene\_cluster | 28 | 83.8372093023 | 222 | 2.7e-57 |
| AJW76710.1 | DsaH | BGC0001196 | Desotamide\_biosynthetic\_gene\_cluster | 28 | 74.1860465116 | 221 | 3.5e-57 |
| EDY47118.1 | N-(5-amino-5-carboxypentanoyl)-L-cysteinyl-D-\_valine\_synthase | BGC0000319 | Cephamycin\_C\_biosynthetic\_gene\_cluster | 28 | 81.3953488372 | 221 | 4.5e-57 |
| BAC67536.1 | arthrofactin\_synthetase\_C | BGC0000305 | Arthrofactin\_biosynthetic\_gene\_cluster | 27 | 79.8837209302 | 220 | 5.9e-57 |
| ABR12615.1 | ACV\_synthetase | BGC0000405 | Penicillin\_biosynthetic\_gene\_cluster | 26 | 81.0465116279 | 220 | 5.9e-57 |
| ABA70582.1 | alpha-aminoadypil-cysteinyl-valine\_synthetase | BGC0000404 | Penicillin\_biosynthetic\_gene\_cluster | 26 | 81.0465116279 | 220 | 5.9e-57 |
| AHD05617.1 | putative\_non-ribosomal\_peptide\_ligase\_domain\_protein | BGC0001033 | Paenilamicin\_biosynthetic\_gene\_cluster | 26 | 81.3953488372 | 220 | 1e-56 |
| AAO23333.1 | NcpA | BGC0000397 | Nostocyclopeptide\_biosynthetic\_gene\_cluster | 26 | 80.4651162791 | 219 | 1.3e-56 |
| AAU34203.1 | mannopeptimycin\_peptide\_synthetase\_MppB | BGC0000388 | Mannopeptimycin\_biosynthetic\_gene\_cluster | 26 | 82.2093023256 | 219 | 1.3e-56 |
| CCJ67648.1 | JagD | BGC0001127 | Jagaricin\_biosynthetic\_gene\_cluster | 28 | 77.3255813953 | 219 | 1.7e-56 |
| AAO56329.1 | non-ribosomal\_peptide\_synthetase\_SyfB | BGC0000435 | Syringafactin\_biosynthetic\_gene\_cluster | 28 | 80.9302325581 | 218 | 2.2e-56 |
| AEZ51520.1 | PmxE | BGC0001153 | Polymyxin\_biosynthetic\_gene\_cluster | 28 | 78.8372093023 | 218 | 3.8e-56 |
| ABV79985.1 | ApnA | BGC0000301 | Anabaenopeptin\_biosynthetic\_gene\_cluster | 28 | 73.6046511628 | 217 | 5e-56 |
| AJM89738.1 | PmxE | BGC0001192 | Colistin\_biosynthetic\_gene\_cluster | 26 | 78.6046511628 | 217 | 5e-56 |
| AGN74876.1 | nonribosomal\_peptide\_synthetase | BGC0000459 | Griseoviridin\_/\_viridogrisein\_biosynthetic\_gene\_cluster | 28 | 81.8604651163 | 217 | 5e-56 |
| AAC06347.1 | bacitracin\_synthetase\_2 | BGC0000310 | Bacitracin\_biosynthetic\_gene\_cluster | 27 | 80.5813953488 | 217 | 6.5e-56 |
| sln7 | nonribosomal\_peptide\_synthetase | BGC0001230 | Salinamides\_biosynthetic\_gene\_cluster | 27 | 81.511627907 | 216 | 1.5e-55 |
| ABL74936.1 | TlmX | BGC0001048 | Tallysomycin\_biosynthetic\_gene\_cluster | 28 | 80.1162790698 | 215 | 1.9e-55 |
| ACA97580.1 | PmxE | BGC0000408 | Polymyxin\_biosynthetic\_gene\_cluster | 28 | 78.8372093023 | 215 | 2.5e-55 |
| CAE02633.1 | surfactin\_synthetase\_C | BGC0000433 | Surfactin\_biosynthetic\_gene\_cluster | 27 | 85.6976744186 | 214 | 5.5e-55 |
| AFJ23826.1 | WLIP\_synthetase\_C | BGC0000462\_c2 | WLIP\_biosynthetic\_gene\_cluster | 27 | 79.6511627907 | 213 | 9.4e-55 |
| ANQ43349.1 | non-ribosomal\_peptide\_synthetase | BGC0001347\_c2 | Poaeamide\_B\_biosynthetic\_gene\_cluster | 27 | 79.6511627907 | 213 | 9.4e-55 |
| AHZ34233.1 | CifB | BGC0000323 | Cichofactin\_biosynthetic\_gene\_cluster | 26 | 82.6744186047 | 212 | 2.7e-54 |
| EFL06867.1 | predicted\_protein | BGC0000300 | Amychelin\_biosynthetic\_gene\_cluster | 28 | 81.3953488372 | 212 | 2.7e-54 |
| ALV86866.1 | Tlo20 | BGC0001406 | Telomycin\_biosynthetic\_gene\_cluster | 27 | 79.1860465116 | 212 | 2.7e-54 |
| AAG02355.1 | peptide\_synthetase\_NRPS9-8 | BGC0000963 | Bleomycin\_biosynthetic\_gene\_cluster | 27 | 80.0 | 212 | 2.7e-54 |
| CCJ67639.1 | TaaD | BGC0000447 | Tolaasin\_biosynthetic\_gene\_cluster | 27 | 77.0930232558 | 211 | 3.6e-54 |
| CDG17982.1 | Non-ribosomal\_peptide\_synthetase | BGC0000464 | Xenoamicins\_biosynthetic\_gene\_cluster | 26 | 81.3953488372 | 211 | 3.6e-54 |
| ABS74206.1 | FenD | BGC0001095 | Fengycin\_biosynthetic\_gene\_cluster | 26 | 80.9302325581 | 211 | 4.7e-54 |
| AEA30273.1 | peptide\_synthetase | BGC0000429 | Skyllamycin\_biosynthetic\_gene\_cluster | 27 | 79.8837209302 | 211 | 4.7e-54 |
| AEW31020.1 | plipastatin\_synthetase | BGC0000407 | Plipastatin\_biosynthetic\_gene\_cluster | 27 | 75.5813953488 | 211 | 4.7e-54 |
| ABV56588.1 | KtzH | BGC0000378 | Kutznerides\_biosynthetic\_gene\_cluster | 28 | 78.2558139535 | 210 | 6.1e-54 |
| AGE25480.1 | amino\_acid\_adenylation\_protein | BGC0001208\_c2 | Poaeamide\_biosynthetic\_gene\_cluster | 26 | 79.3023255814 | 210 | 8e-54 |
| ACZ55945.1 | non-ribosomal\_peptide\_synthetase | BGC0000302 | Anabaenopeptin\_biosynthetic\_gene\_cluster | 29 | 74.3023255814 | 210 | 8e-54 |
| AEH59099.1 | amino\_acid\_adenylation\_domain-containing\_protein/NRPS | BGC0000385 | Lysobactin\_biosynthetic\_gene\_cluster | 30 | 72.4418604651 | 210 | 1e-53 |
| AJF34464.1 | Txo2 | BGC0001207 | Teixobactin\_biosynthetic\_gene\_cluster | 27 | 81.1627906977 | 208 | 2.3e-53 |
| CAJ77696.1 | MPS2\_protein | BGC0000363 | Glycopeptidolipid\_biosynthetic\_gene\_cluster | 27 | 80.2325581395 | 208 | 2.3e-53 |
| ALV82356.1 | CDA\_peptide\_synthetase\_I | BGC0001370 | Lipopeptide\_8D1-1\_/\_lipopeptide\_8D1-2\_biosynthetic\_gene\_cluster | 28 | 77.0930232558 | 208 | 4e-53 |
| CDG17985.1 | Putative\_Ornithine\_racemase\_(fragment) | BGC0000464 | Xenoamicins\_biosynthetic\_gene\_cluster | 28 | 69.8837209302 | 207 | 5.2e-53 |
| AFJ23819.1 | WLIP\_synthetase\_A | BGC0000462 | WLIP\_biosynthetic\_gene\_cluster | 27 | 78.8372093023 | 207 | 6.8e-53 |
| AFH75321.1 | nonribosomal\_peptide\_synthetase | BGC0000425 | Sessilin\_biosynthetic\_gene\_cluster | 29 | 75.9302325581 | 207 | 6.8e-53 |
| KPN93065.1 | NunD | BGC0001416 | Nunapeptin\_/\_nunamycin\_biosynthetic\_gene\_cluster | 27 | 75.5813953488 | 207 | 6.8e-53 |
| ABD65958.1 | nonribosomal\_peptide\_synthetase | BGC0000341 | Enduracidin\_biosynthetic\_gene\_cluster | 30 | 65.9302325581 | 205 | 2e-52 |
| AEW31022.1 | plipastatin\_synthetase | BGC0000407 | Plipastatin\_biosynthetic\_gene\_cluster | 26 | 82.9069767442 | 205 | 2e-52 |
| AAZ55900.1 | non-ribosomal\_peptide\_synthase:Amino\_acid\_adenylation | BGC0000359 | Fuscachelin\_biosynthetic\_gene\_cluster | 26 | 79.7674418605 | 205 | 2e-52 |
| AAY93445.1 | non-ribosomal\_peptide\_synthetase\_PvdL | BGC0000413 | Pyoverdine\_biosynthetic\_gene\_cluster | 27 | 84.0697674419 | 205 | 2e-52 |
| CDG17980.1 | Putative\_Ornithine\_racemase\_(fragment) | BGC0000464 | Xenoamicins\_biosynthetic\_gene\_cluster | 27 | 76.976744186 | 205 | 2e-52 |
| CCJ67646.1 | JagB | BGC0001127 | Jagaricin\_biosynthetic\_gene\_cluster | 29 | 70.3488372093 | 205 | 2.6e-52 |
| AAY93354.1 | non-ribosomal\_peptide\_synthetase\_PvdD | BGC0000413 | Pyoverdine\_biosynthetic\_gene\_cluster | 26 | 80.6976744186 | 204 | 4.4e-52 |
| EFE73308.1 | non-ribosomal\_peptide\_synthetase | BGC0000431 | Stenothricin\_biosynthetic\_gene\_cluster | 29 | 80.6976744186 | 204 | 5.7e-52 |
| BAH43870.1 | putative\_linear\_pentadecapeptide\_gramicidin\_synthetase\_LgrB | BGC0000367 | Gramicidin\_biosynthetic\_gene\_cluster | 26 | 81.7441860465 | 204 | 5.7e-52 |
| CCM44337.1 | putative\_nonribosomal\_peptide\_synthetase | BGC0001056 | Zeamine\_biosynthetic\_gene\_cluster | 28 | 82.7906976744 | 203 | 7.5e-52 |
| WP\_010369430.1 | Non-ribosomal\_peptide\_synthase | BGC0000314 | Bromoalterochromides\_biosynthetic\_gene\_cluster | 23 | 105.11627907 | 203 | 9.8e-52 |
| AHZ34243.1 | CipF | BGC0001389 | Cichopeptin\_biosynthetic\_gene\_cluster | 29 | 77.3255813953 | 202 | 2.2e-51 |
| AGE25479.1 | putative\_non-ribosomal\_peptide\_synthetase | BGC0001208\_c2 | Poaeamide\_biosynthetic\_gene\_cluster | 26 | 81.0465116279 | 202 | 2.2e-51 |
| AEP18656.1 | WAPS1 | BGC0000461 | WAP-8294A2\_(lotilibcin)\_biosynthetic\_gene\_cluster | 28 | 73.023255814 | 201 | 2.8e-51 |
| AHH53506.1 | non-ribosomal\_peptide\_synthetase | BGC0000439 | Taromycin\_biosynthetic\_gene\_cluster | 27 | 79.8837209302 | 201 | 3.7e-51 |
| AMK48226.1 | nonribosomal\_peptide\_synthetase | BGC0001351 | Cahuitamycins\_biosynthetic\_gene\_cluster | 27 | 80.2325581395 | 201 | 3.7e-51 |
| AAU34202.1 | mannopeptimycin\_peptide\_synthetase\_MppA | BGC0000388 | Mannopeptimycin\_biosynthetic\_gene\_cluster | 26 | 76.511627907 | 201 | 4.9e-51 |
| ABX37382.1 | amino\_acid\_adenylation\_domain\_protein | BGC0000984 | Delftibactin\_biosynthetic\_gene\_cluster | 27 | 79.7674418605 | 201 | 4.9e-51 |
| AAT09804.1 | NocA | BGC0000395 | Nocardicin\_A\_biosynthetic\_gene\_cluster | 28 | 80.6976744186 | 201 | 4.9e-51 |
| AID65225.1 | nonribosomal\_peptide\_synthetase | BGC0000335 | Cystomanamides\_biosynthetic\_gene\_cluster | 28 | 73.6046511628 | 200 | 6.3e-51 |
| AAY93356.2 | non-ribosomal\_peptide\_synthetase\_PvdI | BGC0000413 | Pyoverdine\_biosynthetic\_gene\_cluster | 25 | 81.3953488372 | 200 | 6.3e-51 |
| ANQ43348.1 | non-ribosomal\_peptide\_synthetase | BGC0001347\_c2 | Poaeamide\_B\_biosynthetic\_gene\_cluster | 27 | 69.6511627907 | 200 | 6.3e-51 |
| AAS98786.1 | JamO | BGC0001001 | Jamaicamide\_biosynthetic\_gene\_cluster | 26 | 76.2790697674 | 200 | 8.3e-51 |
| CAE02630.1 | surfactin\_synthetase\_A | BGC0000433 | Surfactin\_biosynthetic\_gene\_cluster | 28 | 83.7209302326 | 200 | 8.3e-51 |
| CCJ67645.1 | JagA | BGC0001127 | Jagaricin\_biosynthetic\_gene\_cluster | 29 | 70.6976744186 | 199 | 1.4e-50 |
| ABH06368.2 | MassB | BGC0000389 | Massetolide\_A\_biosynthetic\_gene\_cluster | 26 | 78.2558139535 | 199 | 1.8e-50 |
| AET98906.1 | putative\_non-ribosomal\_peptide\_synthetase | BGC0000415 | Quinomycin\_biosynthetic\_gene\_cluster | 27 | 77.2093023256 | 198 | 2.4e-50 |
| AHZ34232.1 | CifA | BGC0000323 | Cichofactin\_biosynthetic\_gene\_cluster | 27 | 77.3255813953 | 198 | 3.1e-50 |
| ACA97576.1 | PmxA | BGC0000408 | Polymyxin\_biosynthetic\_gene\_cluster | 25 | 81.0465116279 | 197 | 7e-50 |
| AIW82282.1 | PuwE | BGC0001125 | Puwainaphycins\_biosynthetic\_gene\_cluster | 26 | 80.5813953488 | 196 | 9.2e-50 |
| CAM02313.1 | putative\_non-ribosomal\_peptide\_synthetase | BGC0000349 | Erythrochelin\_biosynthetic\_gene\_cluster | 27 | 79.5348837209 | 196 | 9.2e-50 |
| ABW17375.1 | PsoA | BGC0000411 | Putisolvin\_biosynthetic\_gene\_cluster | 27 | 78.1395348837 | 196 | 1.2e-49 |
| AID65224.1 | nonribosomal\_peptide\_synthetase | BGC0000335 | Cystomanamides\_biosynthetic\_gene\_cluster | 25 | 80.1162790698 | 196 | 1.2e-49 |
| ADD82940.1 | Bat2 | BGC0001099 | Kalimantacin\_/\_batumin\_biosynthetic\_gene\_cluster | 27 | 78.3720930233 | 195 | 2e-49 |
| AHD05618.1 | putative\_non-ribosomal\_peptide\_ligase\_domain\_protein | BGC0001033 | Paenilamicin\_biosynthetic\_gene\_cluster | 25 | 79.0697674419 | 195 | 2e-49 |
| ACA97577.1 | PmxB | BGC0000408 | Polymyxin\_biosynthetic\_gene\_cluster | 27 | 75.2325581395 | 195 | 2.7e-49 |
| ACM79812.1 | ZmaQ | BGC0001059 | Zwittermycin\_A\_biosynthetic\_gene\_cluster | 25 | 85.1162790698 | 195 | 2.7e-49 |
| BAH04162.1 | trsJ | BGC0000450 | Triostin\_A\_biosynthetic\_gene\_cluster | 26 | 74.4186046512 | 195 | 2.7e-49 |
| ABP57748.1 | DepD | BGC0000993 | FK228\_biosynthetic\_gene\_cluster | 26 | 79.0697674419 | 194 | 4.5e-49 |
| CAJ77716.1 | Mps2\_protein | BGC0000364 | Glycopeptidolipid\_biosynthetic\_gene\_cluster | 27 | 80.6976744186 | 194 | 5.9e-49 |
| ABF87031.1 | non-ribosomal\_peptide\_synthetase/polyketide\_synthase | BGC0000393 | Myxoprincomide\_biosynthetic\_gene\_cluster | 28 | 85.2325581395 | 194 | 5.9e-49 |
| AJM89734.1 | PmxB | BGC0001192 | Colistin\_biosynthetic\_gene\_cluster | 27 | 75.4651162791 | 194 | 5.9e-49 |
| CAK15815.1 | putative\_non\_ribosomal\_peptide\_synthetase | BGC0000344 | Entolysin\_biosynthetic\_gene\_cluster | 27 | 71.511627907 | 193 | 7.7e-49 |
| ADG27358.1 | peptide\_synthetase | BGC0000296 | Actinomycin\_biosynthetic\_gene\_cluster | 27 | 77.6744186047 | 193 | 7.7e-49 |
| AFH75328.1 | nonribosomal\_peptide\_synthetase | BGC0000398 | Orfamide\_biosynthetic\_gene\_cluster | 26 | 78.7209302326 | 193 | 1e-48 |
| KPN90369.1 | NunE | BGC0001416 | Nunapeptin\_/\_nunamycin\_biosynthetic\_gene\_cluster | 27 | 77.4418604651 | 193 | 1.3e-48 |
| AAX31558.1 | peptide\_synthetase\_2 | BGC0000336 | Daptomycin\_biosynthetic\_gene\_cluster | 26 | 79.0697674419 | 193 | 1.3e-48 |
| CAJ96470.1 | Non-ribosomal\_peptide\_synthetase | BGC0000330 | Cupriachelin\_biosynthetic\_gene\_cluster | 27 | 80.6976744186 | 191 | 3.8e-48 |
| AEZ51517.1 | PmxB | BGC0001153 | Polymyxin\_biosynthetic\_gene\_cluster | 27 | 70.4651162791 | 191 | 5e-48 |
| AHD05621.1 | non-ribosomal\_peptide\_ligase\_domain\_protein | BGC0001033 | Paenilamicin\_biosynthetic\_gene\_cluster | 26 | 83.7209302326 | 191 | 5e-48 |
| AHZ34241.1 | CipD | BGC0001389 | Cichopeptin\_biosynthetic\_gene\_cluster | 28 | 79.7674418605 | 190 | 6.6e-48 |
| WP\_028678149.1 | non-ribosomal\_peptide\_synthetase | BGC0001228 | Retimycin\_biosynthetic\_gene\_cluster | 26 | 79.5348837209 | 190 | 1.1e-47 |
| CAJ46692.1 | non\_ribosomal\_peptide\_synthase | BGC0000969 | Chondramide\_biosynthetic\_gene\_cluster | 30 | 62.0930232558 | 189 | 1.5e-47 |
| BAI63288.1 | putative\_non-ribosomal\_peptide\_synthetase | BGC0000434 | SW-163\_biosynthetic\_gene\_cluster | 27 | 71.2790697674 | 189 | 1.5e-47 |
| AGZ15458.1 | putative\_non-ribosomal\_peptide\_synthetase | BGC0001036 | Polyoxypeptin\_biosynthetic\_gene\_cluster | 25 | 79.8837209302 | 189 | 1.5e-47 |
| BAH43869.1 | linear\_pentadecapeptide\_gramicidin\_synthetase\_LgrA | BGC0000367 | Gramicidin\_biosynthetic\_gene\_cluster | 24 | 80.5813953488 | 189 | 1.5e-47 |
| AED90003.1 | non-ribosomal\_peptide\_synthetase\_ThaB | BGC0000443 | Thanamycin\_biosynthetic\_gene\_cluster | 25 | 77.6744186047 | 189 | 1.9e-47 |
| CAD32904.2 | non-ribosomal\_peptide\_synthetase\_A | BGC0000354 | Friulimicin\_biosynthetic\_gene\_cluster | 27 | 79.6511627907 | 188 | 2.5e-47 |
| AJW76709.1 | DsaG | BGC0001196 | Desotamide\_biosynthetic\_gene\_cluster | 28 | 81.0465116279 | 188 | 4.3e-47 |
| EJK79842.1 | amino\_acid\_adenylation\_enzyme/thioester\_reductase\_family\_protein | BGC0000436 | Syringolin\_A\_biosynthetic\_gene\_cluster | 25 | 80.8139534884 | 187 | 5.6e-47 |
| AGZ15459.1 | putative\_non-ribosomal\_peptide\_synthetase | BGC0001036 | Polyoxypeptin\_biosynthetic\_gene\_cluster | 25 | 80.4651162791 | 187 | 7.3e-47 |
| AAY37647.1 | Amino\_acid\_adenylation | BGC0000437 | Syringomycin\_biosynthetic\_gene\_cluster | 27 | 77.9069767442 | 187 | 7.3e-47 |
| AHH53508.1 | non-ribosomal\_peptide\_synthetase | BGC0000439 | Taromycin\_biosynthetic\_gene\_cluster | 28 | 67.6744186047 | 187 | 7.3e-47 |
| CDG17987.1 | Putative\_Ornithine\_racemase\_(fragment) | BGC0000464 | Xenoamicins\_biosynthetic\_gene\_cluster | 26 | 75.4651162791 | 187 | 7.3e-47 |
| ABG94125.1 | non-ribosomal\_peptide\_synthetase | BGC0000417 | Rhodochelin\_biosynthetic\_gene\_cluster | 26 | 78.9534883721 | 186 | 9.5e-47 |
| ADJ63842.1 | non-ribosomal\_peptide\_synthetase\_protein | BGC0000424 | Serobactins\_biosynthetic\_gene\_cluster | 27 | 69.8837209302 | 185 | 2.1e-46 |
| WP\_010369428.1 | phenylalanine\_racemase | BGC0000314 | Bromoalterochromides\_biosynthetic\_gene\_cluster | 24 | 94.3023255814 | 185 | 2.1e-46 |
| AIS24862.1 | Dst18 | BGC0001147 | Distamycin\_biosynthetic\_gene\_cluster | 30 | 69.7674418605 | 185 | 2.1e-46 |
| ABC94347.1 | vicibactin\_biosynthesis\_non-ribosomal\_peptide\_synthase\_protein | BGC0000457 | Vicibactin\_biosynthetic\_gene\_cluster | 25 | 82.7906976744 | 185 | 2.1e-46 |
| AHD05627.1 | putative\_non-ribosomal\_peptide\_ligase\_domain\_protein | BGC0001033 | Paenilamicin\_biosynthetic\_gene\_cluster | 26 | 73.2558139535 | 185 | 2.8e-46 |
| CAJ88610.1 | putative\_non-ribosomal\_peptide\_synthetase | BGC0000327 | Congocidine\_biosynthetic\_gene\_cluster | 30 | 69.1860465116 | 185 | 2.8e-46 |
| ANQ43347.1 | non-ribosomal\_peptide\_synthetase | BGC0001347 | Poaeamide\_B\_biosynthetic\_gene\_cluster | 26 | 75.2325581395 | 185 | 2.8e-46 |
| ACM68690.1 | AerG1 | BGC0000298 | Aeruginosin\_biosynthetic\_gene\_cluster | 24 | 81.7441860465 | 185 | 3.6e-46 |
| ADN26252.1 | peptide\_synthetase | BGC0000951 | Pacidamycin\_biosynthetic\_gene\_cluster | 27 | 73.6046511628 | 185 | 3.6e-46 |
| CAB53322.1 | putative\_peptide\_synthetase | BGC0000325 | Coelichelin\_biosynthetic\_gene\_cluster | 26 | 78.1395348837 | 184 | 4.7e-46 |
| AHI59110.1 | locillomycin\_synthase\_C | BGC0001005 | Locillomycin\_biosynthetic\_gene\_cluster | 25 | 77.6744186047 | 184 | 4.7e-46 |
| ADA69239.2 | trans-AT\_hybrid\_polyketide\_synthase-NRPS | BGC0001071 | Nosperin\_biosynthetic\_gene\_cluster | 25 | 76.7441860465 | 184 | 4.7e-46 |
| AKP45395.1 | CysG | BGC0001413 | Cystobactamide\_biosynthetic\_gene\_cluster | 28 | 69.7674418605 | 184 | 6.1e-46 |
| AAF00960.1 | McyA | BGC0001017 | Microcystin\_biosynthetic\_gene\_cluster | 28 | 60.6976744186 | 183 | 8e-46 |
| AAR87760.2 | ZmaK | BGC0001059 | Zwittermycin\_A\_biosynthetic\_gene\_cluster | 23 | 81.7441860465 | 183 | 1e-45 |
| ABL74940.1 | TlmVI | BGC0001048 | Tallysomycin\_biosynthetic\_gene\_cluster | 25 | 80.2325581395 | 183 | 1.4e-45 |
| CAY48789.1 | putative\_non-ribosomal\_peptide\_synthetase | BGC0001312 | Viscosin\_biosynthetic\_gene\_cluster | 27 | 77.2093023256 | 182 | 1.8e-45 |
| ERG36783.1 | phenylalanine\_racemase | BGC0000299 | Alterochromides\_biosynthetic\_gene\_cluster | 25 | 79.7674418605 | 182 | 2.3e-45 |
| CAD29794.1 | peptide\_synthetase | BGC0001015 | Microcystin\_biosynthetic\_gene\_cluster | 26 | 78.023255814 | 182 | 2.3e-45 |
| AAU39360.1 | lichenysin\_synthase\_LchAB | BGC0000381 | Lichenysin\_biosynthetic\_gene\_cluster | 26 | 79.3023255814 | 182 | 2.3e-45 |
| CBJ79915.1 | putative\_Phenylalanine\_racemase\_(ATP-hydrolyzing) | BGC0001133 | Taxlllaid\_biosynthetic\_gene\_cluster | 25 | 80.3488372093 | 182 | 2.3e-45 |
| CAK15814.1 | putative\_non-ribosomal\_peptide\_synthetase,\_terminal\_component | BGC0000344 | Entolysin\_biosynthetic\_gene\_cluster | 27 | 75.8139534884 | 182 | 2.3e-45 |
| CAE52339.1 | Polyketide\_non-ribosomal\_peptide\_synthase | BGC0001088 | Albicidin\_biosynthetic\_gene\_cluster | 26 | 81.511627907 | 181 | 5.2e-45 |
| AGE26255.1 | putative\_non-ribosomal\_peptide\_synthetase | BGC0001208 | Poaeamide\_biosynthetic\_gene\_cluster | 25 | 72.4418604651 | 181 | 5.2e-45 |
| CAD29797.1 | peptide\_synthetase | BGC0001015 | Microcystin\_biosynthetic\_gene\_cluster | 24 | 81.2790697674 | 181 | 5.2e-45 |
| AAZ03551.1 | McnB | BGC0000332 | Cyanopeptolin\_biosynthetic\_gene\_cluster | 24 | 84.7674418605 | 181 | 5.2e-45 |
| AAX31557.1 | peptide\_synthetase\_1 | BGC0000336 | Daptomycin\_biosynthetic\_gene\_cluster | 26 | 78.6046511628 | 180 | 6.8e-45 |
| CBZ42143.1 | putative\_non-ribosomal\_peptide\_synthetase | BGC0001117 | Himastatin\_biosynthetic\_gene\_cluster | 27 | 72.6744186047 | 180 | 8.9e-45 |
| AAZ23075.1 | peptide\_synthetase | BGC0000291 | A54145\_biosynthetic\_gene\_cluster | 26 | 84.3023255814 | 180 | 8.9e-45 |
| ACU36654.1 | amino\_acid\_adenylation\_domain\_protein | BGC0000392 | Mirubactin\_biosynthetic\_gene\_cluster | 27 | 77.9069767442 | 180 | 1.2e-44 |
| BAH22763.1 | nonribosomal\_peptide\_synthetase | BGC0001018 | Micropeptin\_biosynthetic\_gene\_cluster | 24 | 81.511627907 | 179 | 1.5e-44 |
| AOA33121.1 | nonribosomal\_peptide\_synthetase | BGC0001346 | Bananamides\_biosynthetic\_gene\_cluster | 26 | 78.3720930233 | 178 | 2.6e-44 |
| ADY76667.1 | non-ribosomal\_peptide\_synthetase | BGC0000950 | Napsamycin\_biosynthetic\_gene\_cluster | 26 | 73.9534883721 | 178 | 2.6e-44 |
| AAC06348.1 | bacitracin\_synthetase\_3 | BGC0000310 | Bacitracin\_biosynthetic\_gene\_cluster | 25 | 85.1162790698 | 178 | 3.4e-44 |
| CBJ79916.1 | putative\_Ornithine\_racemase | BGC0001133 | Taxlllaid\_biosynthetic\_gene\_cluster | 24 | 79.7674418605 | 178 | 3.4e-44 |
| ALG65314.1 | Cal22 | BGC0001297 | WS9326\_biosynthetic\_gene\_cluster | 27 | 72.0930232558 | 178 | 3.4e-44 |
| AGN74885.1 | nonribosomal\_peptide\_synthetase | BGC0000459 | Griseoviridin\_/\_viridogrisein\_biosynthetic\_gene\_cluster | 27 | 79.0697674419 | 178 | 4.4e-44 |
| ABD14712.1 | CesB | BGC0000320 | Cereulide\_biosynthetic\_gene\_cluster | 23 | 80.5813953488 | 177 | 7.5e-44 |
| BAE98155.1 | putative\_non-ribosomal\_peptide\_synthetase | BGC0000339 | Echinomycin\_biosynthetic\_gene\_cluster | 26 | 75.9302325581 | 175 | 2.2e-43 |
| AAK81824.1 | peptide\_synthetase | BGC0000326 | Complestatin\_biosynthetic\_gene\_cluster | 26 | 78.7209302326 | 175 | 2.9e-43 |
| CAG29032.1 | nonribosomal\_peptide\_synthetase\_(modules\_3\_to\_6) | BGC0001023 | Myxochromide\_biosynthetic\_gene\_cluster | 26 | 79.8837209302 | 175 | 2.9e-43 |
| CAD70194.1 | non-ribosomal\_peptide\_synthetase | BGC0001047 | Syringolin\_A\_biosynthetic\_gene\_cluster | 27 | 70.4651162791 | 175 | 3.7e-43 |
| ACG60773.1 | NRPS(C/A/PCP) | BGC0001058 | Zorbamycin\_biosynthetic\_gene\_cluster | 27 | 69.6511627907 | 175 | 3.7e-43 |
| AGC09519.1 | AMP-dependent\_synthetase/ligase | BGC0001183 | Lobophorin\_biosynthetic\_gene\_cluster | 29 | 74.8837209302 | 175 | 3.7e-43 |
| AEP18655.1 | WAPS2 | BGC0000461 | WAP-8294A2\_(lotilibcin)\_biosynthetic\_gene\_cluster | 24 | 82.4418604651 | 174 | 4.9e-43 |
| AEW31015.1 | plipastatin\_synthetase | BGC0000407 | Plipastatin\_biosynthetic\_gene\_cluster | 26 | 80.5813953488 | 174 | 4.9e-43 |
| AHH53507.1 | non-ribosomal\_peptide\_synthetase | BGC0000439 | Taromycin\_biosynthetic\_gene\_cluster | 27 | 79.8837209302 | 174 | 6.4e-43 |
| BAC67534.2 | arthrofactin\_synthetase\_A | BGC0000305 | Arthrofactin\_biosynthetic\_gene\_cluster | 24 | 80.0 | 173 | 8.3e-43 |
| AAF08796.1 | MycB | BGC0001103 | Mycosubtilin\_biosynthetic\_gene\_cluster | 24 | 80.8139534884 | 173 | 1.4e-42 |
| CCM44336.1 | putative\_nonribosomal\_peptide\_synthetase | BGC0001056 | Zeamine\_biosynthetic\_gene\_cluster | 25 | 82.2093023256 | 173 | 1.4e-42 |
| CBZ42140.1 | non-ribosomal\_peptide\_synthetase | BGC0001117 | Himastatin\_biosynthetic\_gene\_cluster | 28 | 70.2325581395 | 173 | 1.4e-42 |
| ABP73646.1 | SalB | BGC0000145 | Salinosporamide\_A\_biosynthetic\_gene\_cluster | 28 | 67.5581395349 | 172 | 1.8e-42 |
| WP\_010369425.1 | ornithine\_racemase | BGC0000314 | Bromoalterochromides\_biosynthetic\_gene\_cluster | 24 | 75.6976744186 | 172 | 1.8e-42 |
| ERG36782.1 | Ornithine\_racemase | BGC0000299 | Alterochromides\_biosynthetic\_gene\_cluster | 24 | 75.6976744186 | 172 | 1.8e-42 |
| ABP53497.1 | amino\_acid\_adenylation\_domain | BGC0001041 | Salinosporamide\_biosynthetic\_gene\_cluster | 28 | 67.5581395349 | 172 | 1.8e-42 |
| KPN93064.1 | NupB | BGC0001416 | Nunapeptin\_/\_nunamycin\_biosynthetic\_gene\_cluster | 26 | 78.023255814 | 172 | 2.4e-42 |
| EAU36744.1 | predicted\_protein | BGC0000292 | Acetylaranotin\_biosynthetic\_gene\_cluster | 26 | 73.488372093 | 170 | 1.2e-41 |
| CAE02631.1 | surfactin\_synthetase\_B | BGC0000433 | Surfactin\_biosynthetic\_gene\_cluster | 27 | 68.2558139535 | 169 | 2e-41 |
| AAY91419.3 | non-ribosomal\_peptide\_synthetase\_OfaA | BGC0000399 | Orfamide\_biosynthetic\_gene\_cluster | 25 | 77.6744186047 | 166 | 1.3e-40 |
| ABL74939.1 | TlmVII | BGC0001048 | Tallysomycin\_biosynthetic\_gene\_cluster | 27 | 67.6744186047 | 166 | 1.3e-40 |
| CAQ71829.1 | non\_ribosomal\_peptide\_synthase,\_antibiotic\_synthesis;\_contains\_4\_condensation\_domains,\_3\_AMP-acid\_ligases\_II\_domains,\_3\_PP-binding,\_Phosphopantetheine\_attachment\_site\_and\_a\_putative\_thioesterase\_domain | BGC0001189 | Taiwachelin\_biosynthetic\_gene\_cluster | 26 | 69.4186046512 | 165 | 3.9e-40 |
| CAE52334.1 | non-ribosomal\_peptide\_synthase | BGC0001088 | Albicidin\_biosynthetic\_gene\_cluster | 25 | 80.3488372093 | 165 | 3.9e-40 |
| AFK57219.1 | DidH | BGC0000985 | Didemnin\_biosynthetic\_gene\_cluster | 27 | 70.9302325581 | 165 | 3.9e-40 |
| ABR67750.1 | CmnG | BGC0000316 | Capreomycin\_biosynthetic\_gene\_cluster | 25 | 72.9069767442 | 165 | 3.9e-40 |
| AAT70101.1 | CurF | BGC0001165 | Curacin\_biosynthetic\_gene\_cluster | 24 | 77.7906976744 | 164 | 5e-40 |
| AEE88284.1 | CurF | BGC0000976 | Curacin\_biosynthetic\_gene\_cluster | 24 | 77.7906976744 | 164 | 5e-40 |
| CAJ34382.1 | NRPS\_protein | BGC0000445 | Thiocoraline\_biosynthetic\_gene\_cluster | 26 | 78.488372093 | 164 | 6.6e-40 |
| AEC14349.1 | nonribosomal\_peptide\_synthetase | BGC0000377 | Koranimine\_biosynthetic\_gene\_cluster | 25 | 72.0930232558 | 163 | 8.6e-40 |
| CAC17499.1 | putative\_non-ribosomal\_peptide\_synthase | BGC0000324 | Coelibactin\_biosynthetic\_gene\_cluster | 27 | 71.2790697674 | 163 | 8.6e-40 |
| ABA59547.1 | Vlm1 | BGC0000453 | Valinomycin\_biosynthetic\_gene\_cluster | 25 | 80.9302325581 | 163 | 1.1e-39 |
| EFG10344.1 | Non-ribosomal\_peptide\_synthetase | BGC0000373 | Holomycin\_biosynthetic\_gene\_cluster | 27 | 73.488372093 | 163 | 1.1e-39 |
| ABG00046.1 | non-ribosomal\_peptide\_synthetase | BGC0000387 | Mangotoxin\_biosynthetic\_gene\_cluster | 27 | 63.6046511628 | 163 | 1.1e-39 |
| ADI59531.1 | CorI | BGC0001091 | Corallopyronin\_biosynthetic\_gene\_cluster | 28 | 72.4418604651 | 163 | 1.5e-39 |
| ABX37383.1 | amino\_acid\_adenylation\_domain\_protein | BGC0000984 | Delftibactin\_biosynthetic\_gene\_cluster | 25 | 81.1627906977 | 162 | 1.9e-39 |
| AIG79243.1 | Hypothetical\_protein | BGC0000419 | Ristomycin\_A\_biosynthetic\_gene\_cluster | 25 | 81.0465116279 | 161 | 5.6e-39 |
| AAZ23077.1 | peptide\_synthetase | BGC0000291 | A54145\_biosynthetic\_gene\_cluster | 24 | 82.4418604651 | 160 | 1.2e-38 |
| ABV56585.1 | KtzE | BGC0000378 | Kutznerides\_biosynthetic\_gene\_cluster | 25 | 74.6511627907 | 159 | 1.6e-38 |
| AIE77057.1 | peptide\_synthetase | BGC0000418 | Ristocetin\_biosynthetic\_gene\_cluster | 25 | 80.5813953488 | 159 | 1.6e-38 |
| CBD77746.1 | non-ribosomal\_peptide\_synthetase/polyketide\_synthase | BGC0000974 | Crocacin\_biosynthetic\_gene\_cluster | 26 | 69.5348837209 | 158 | 4.7e-38 |
| CAE14171.1 |  | BGC0000383 | Luminmycin\_biosynthetic\_gene\_cluster | 25 | 83.9534883721 | 158 | 4.7e-38 |
| ADZ45324.1 | non-ribosomal\_peptide\_synthetase | BGC0001020 | Muraymycin\_biosynthetic\_gene\_cluster | 26 | 79.7674418605 | 158 | 4.7e-38 |
| ABD65960.1 | nonribosomal\_peptide\_synthetase | BGC0000341 | Enduracidin\_biosynthetic\_gene\_cluster | 26 | 70.2325581395 | 157 | 6.2e-38 |
| AHB38511.1 | EpnJ | BGC0000345 | Eponemycin\_biosynthetic\_gene\_cluster | 26 | 74.1860465116 | 157 | 8e-38 |
| CCE28989.1 | non-ribosomal\_peptide\_synthetase | BGC0001365 | Clapurines\_biosynthetic\_gene\_cluster | 27 | 69.6511627907 | 157 | 8e-38 |
| AEA29643.1 | putative\_nonribosomal\_peptide\_synthetase | BGC0000409 | Porothramycin\_biosynthetic\_gene\_cluster | 27 | 69.8837209302 | 154 | 5.2e-37 |
| AAN85506.1 | nonribosomal\_peptide\_synthetase | BGC0001101 | Leinamycin\_biosynthetic\_gene\_cluster | 27 | 67.7906976744 | 154 | 5.2e-37 |
| CBK62746.1 |  | BGC0001115 | Thiomarinol\_biosynthetic\_gene\_cluster | 25 | 67.9069767442 | 153 | 8.9e-37 |
| AKP45399.1 | CysK | BGC0001413 | Cystobactamide\_biosynthetic\_gene\_cluster | 25 | 82.7906976744 | 153 | 8.9e-37 |
| CCJ67647.1 | JagC | BGC0001127 | Jagaricin\_biosynthetic\_gene\_cluster | 28 | 73.8372093023 | 153 | 1.2e-36 |
| CAJ34374.1 | NRPS\_protein | BGC0000445 | Thiocoraline\_biosynthetic\_gene\_cluster | 26 | 69.1860465116 | 152 | 2e-36 |
| AAP92497.1 | nonribosomal\_peptide\_synthetase | BGC0000458 | Viomycin\_biosynthetic\_gene\_cluster | 25 | 72.4418604651 | 152 | 2.6e-36 |
| BAP16689.1 | nonribosomal\_peptide\_synthetase | BGC0000376 | JBIR-34,\_JBIR-35\_biosynthetic\_gene\_cluster | 26 | 77.6744186047 | 151 | 5.8e-36 |
| BAU50944.1 | nonribosomal\_peptide\_synthetase | BGC0001379 | BD-12\_biosynthetic\_gene\_cluster | 28 | 67.7906976744 | 149 | 1.3e-35 |
| AAC32048.1 | FxbC | BGC0000351 | Exochelin\_biosynthetic\_gene\_cluster | 25 | 79.1860465116 | 146 | 1.1e-34 |
| AHB38515.1 | EpnG | BGC0000345 | Eponemycin\_biosynthetic\_gene\_cluster | 24 | 71.3953488372 | 146 | 1.1e-34 |
| CAF32362.1 | putative\_non-ribosomal\_peptide\_synthetase | BGC0000712 | Paromomycin\_biosynthetic\_gene\_cluster | 27 | 66.976744186 | 146 | 1.9e-34 |
| AAO23334.1 | NcpB | BGC0000397 | Nostocyclopeptide\_biosynthetic\_gene\_cluster | 28 | 56.1627906977 | 146 | 1.9e-34 |
| BAE56606.1 |  | BGC0001123 | Aspirochlorine\_biosynthetic\_gene\_cluster | 25 | 66.3953488372 | 145 | 2.4e-34 |
| AFK57215.1 | DidD | BGC0000985 | Didemnin\_biosynthetic\_gene\_cluster | 25 | 81.8604651163 | 144 | 4.1e-34 |
| CBA63680.1 | nonribosomal\_peptide\_synthetase\_NRPS | BGC0000368 | Griseobactin\_biosynthetic\_gene\_cluster | 27 | 70.6976744186 | 144 | 7e-34 |
| ADL64235.1 | aureusimine\_non-ribosomal\_peptide\_synthetase | BGC0000308 | Aureusimine\_biosynthetic\_gene\_cluster | 24 | 75.4651162791 | 144 | 7e-34 |
| AKA59447.1 | non-ribosomal\_peptide\_synthetase | BGC0001203 | Clarexpoxcin\_biosynthetic\_gene\_cluster | 27 | 58.6046511628 | 142 | 2.7e-33 |
| AAS92545.1 | SirP | BGC0001044 | Sirodesmin\_biosynthetic\_gene\_cluster | 29 | 49.8837209302 | 141 | 3.5e-33 |
| ADG27359.1 | peptide\_synthetase | BGC0000296 | Actinomycin\_biosynthetic\_gene\_cluster | 26 | 60.6976744186 | 140 | 7.8e-33 |
| AEI58865.1 | peptide\_synthetase | BGC0000455 | Vancomycin\_biosynthetic\_gene\_cluster | 27 | 57.0930232558 | 139 | 1.3e-32 |
| AEW31019.1 | plipastatin\_synthetase | BGC0000407 | Plipastatin\_biosynthetic\_gene\_cluster | 23 | 80.6976744186 | 139 | 1.7e-32 |
| BAF50727.1 | hybrid\_polyketide\_synthase-non\_ribosomal\_peptide\_synthetase | BGC0001116 | Virginiamycin\_biosynthetic\_gene\_cluster | 27 | 60.8139534884 | 138 | 3e-32 |
| CCP45171.1 | Phenyloxazoline\_synthase\_MbtB\_(phenyloxazoline\_synthetase) | BGC0001021 | Mycobactin\_biosynthetic\_gene\_cluster | 24 | 71.8604651163 | 138 | 3.9e-32 |
| ALU98443.1 | hypothetical\_protein | BGC0001397 | Lidamycin\_biosynthetic\_gene\_cluster | 27 | 61.3953488372 | 137 | 6.6e-32 |
| CAA11794.1 | PCZA363.3 | BGC0000322 | Chloroeremomycin\_biosynthetic\_gene\_cluster | 26 | 56.8604651163 | 136 | 1.1e-31 |
| AKA59436.1 | non-ribosomal\_peptide\_synthetase | BGC0001202 | Landepoxcin\_biosynthetic\_gene\_cluster | 25 | 53.8372093023 | 136 | 1.9e-31 |
| AAL06681.1 | A-domain\_type\_II\_peptide\_synthetase | BGC0000965 | C-1027\_biosynthetic\_gene\_cluster | 30 | 46.511627907 | 133 | 9.5e-31 |
| CAC48360.1 | peptide\_synthetase | BGC0000311 | Balhimycin\_biosynthetic\_gene\_cluster | 26 | 57.9069767442 | 133 | 1.2e-30 |
| ADN43685.1 | DmbS | BGC0001136 | Desmethylbassianin\_biosynthetic\_gene\_cluster | 23 | 74.5348837209 | 132 | 2.1e-30 |
| ABC36203.1 | thiotemplate\_mechanism\_natural\_product\_synthetase | BGC0001102 | Malleilactone\_biosynthetic\_gene\_cluster | 26 | 76.8604651163 | 132 | 2.1e-30 |
| CAN89664.1 | putative\_amino\_acid\_adenylating\_enzyme | BGC0001070 | Kirromycin\_biosynthetic\_gene\_cluster | 25 | 60.3488372093 | 129 | 1.8e-29 |
| ACG60782.1 | NRPS(C/A/PCP/C/A/PCP) | BGC0001058 | Zorbamycin\_biosynthetic\_gene\_cluster | 26 | 79.7674418605 | 128 | 5.2e-29 |
| EAW09117.1 | hybrid\_NRPS/PKS\_enzyme,\_putative | BGC0000983 | Cytochalasin\_biosynthetic\_gene\_cluster | 25 | 71.3953488372 | 127 | 8.9e-29 |
| ALV82447.1 | putative\_non-ribosomal\_peptide\_synthetase | BGC0001382 | Svaricin\_biosynthetic\_gene\_cluster | 25 | 74.1860465116 | 127 | 8.9e-29 |
| AFV27434.1 | indigoidine\_synthase | BGC0000375 | Indigoidine\_biosynthetic\_gene\_cluster | 26 | 48.1395348837 | 125 | 3.4e-28 |
| CDG12865.1 | non-ribosomal\_peptide\_synthetase | BGC0001415 | Althiomycin\_biosynthetic\_gene\_cluster | 25 | 54.6511627907 | 121 | 6.4e-27 |
| AAF19815.1 | MtaG | BGC0001024 | Myxothiazol\_biosynthetic\_gene\_cluster | 29 | 43.3720930233 | 114 | 4.6e-25 |
| AAC32047.1 | FxbB | BGC0000351 | Exochelin\_biosynthetic\_gene\_cluster | 31 | 32.4418604651 | 111 | 5.1e-24 |
| WP\_051700105.1 | non-ribosomal\_peptide\_synthetase | BGC0001368 | Tambromycin\_biosynthetic\_gene\_cluster | 30 | 32.9069767442 | 110 | 1.1e-23 |
| ABX71111.1 | Lct28 | BGC0000238 | Lactonamycin\_biosynthetic\_gene\_cluster | 26 | 64.1860465116 | 109 | 1.9e-23 |
| ALA09365.1 | AMP-dependent\_synthetase\_and\_ligase | BGC0001303 | Lobosamide\_biosynthetic\_gene\_cluster | 26 | 61.0465116279 | 109 | 2.5e-23 |
| CAD89778.1 | MelG\_protein | BGC0001010 | Melithiazol\_biosynthetic\_gene\_cluster | 31 | 28.2558139535 | 105 | 3.6e-22 |
| CAE52342.1 | non-ribosomal\_peptide\_synthase | BGC0001088 | Albicidin\_biosynthetic\_gene\_cluster | 27 | 47.5581395349 | 103 | 1.1e-21 |
| CBJ89771.1 | Non-ribosomal\_peptide\_synthase\_involved\_in\_Xenocoumacin\_synthesis | BGC0001054 | Xenocoumacin\_biosynthetic\_gene\_cluster | 22 | 78.1395348837 | 102 | 1.8e-21 |
| EFY94493.1 | nonribosomal\_peptide\_synthase\_GliP-like,\_putative | BGC0000337 | Destruxins\_biosynthetic\_gene\_cluster | 25 | 48.2558139535 | 101 | 6.8e-21 |
| ABP55217.1 | AMP-dependent\_synthetase\_and\_ligase | BGC0000142 | Salinilactam\_biosynthetic\_gene\_cluster | 25 | 60.9302325581 | 100 | 8.9e-21 |
| ADQ55476.1 | non-ribosomal\_peptide\_synthase\_dimodule | BGC0000350 | ET-743\_biosynthetic\_gene\_cluster | 29 | 31.2790697674 | 92 | 1.9e-18 |
| CAD29795.1 | peptide\_synthetase | BGC0001015 | Microcystin\_biosynthetic\_gene\_cluster | 23 | 74.6511627907 | 84 | 8.6e-16 |
| AAN32979.1 | BarE | BGC0000962 | Barbamide\_biosynthetic\_gene\_cluster | 23 | 59.7674418605 | 84 | 8.6e-16 |
| CAH55637.1 | putative\_L-prolyl-AMP\_ligase | BGC0000258 | Prodigiosin\_biosynthetic\_gene\_cluster | 23 | 59.5348837209 | 81 | 5.6e-15 |
| AAO39105.1 | AdmK | BGC0000956 | Andrimid\_biosynthetic\_gene\_cluster | 26 | 43.7209302326 | 77 | 8.1e-14 |
| AGI61652.1 | long\_chain\_fatty\_acid\_CoA\_ligase | BGC0000095\_c2 | Micromonolactam\_biosynthetic\_gene\_cluster | 31 | 21.511627907 | 76 | 1.8e-13 |
| CAI94718.1 | putative\_CoA\_ligase | BGC0000141 | Rubradirin\_biosynthetic\_gene\_cluster | 26 | 48.3720930233 | 72 | 2e-12 |
| CBG75492.1 | putative\_NRPS/siderophore\_biosynthesis\_protein | BGC0000423 | Scabichelin\_biosynthetic\_gene\_cluster | 31 | 21.2790697674 | 69 | 2.9e-11 |
| CBW45681.1 | putative\_amino\_acid\_adenyltransferase | BGC0000952\_c2 | Pristinamycin\_biosynthetic\_gene\_cluster | 25 | 43.9534883721 | 68 | 4.9e-11 |
| CBA11557.1 | non-ribosomal\_peptide\_synthetase | BGC0001046 | Streptolydigin\_biosynthetic\_gene\_cluster | 26 | 26.0465116279 | 64 | 5.4e-10 |
| BAE98156.1 | putative\_non-ribosomal\_peptide\_synthetase | BGC0000339 | Echinomycin\_biosynthetic\_gene\_cluster | 30 | 20.8139534884 | 64 | 7.1e-10 |
| AKD43499.1 | Acyl-CoA\_ligase | BGC0001409 | Dutomycin\_biosynthetic\_gene\_cluster | 24 | 48.8372093023 | 60 | 1e-08 |
| ctg1\_orf10 |  | BGC0000321 | Chaetocin\_biosynthetic\_gene\_cluster | 25 | 43.8372093023 | 56 | 1.9e-07 |
